# A case report: corheart 6 biventricular assist device therapy for end-stage heart failure in dilated cardiomyopathy

**DOI:** 10.3389/fcvm.2025.1677940

**Published:** 2025-10-28

**Authors:** Qiuju Ding, Cheng Chen, Zhenjun Xu, Ning Zhang, Jun Pan, Min Ge

**Affiliations:** Department of Cardio-thoracic Surgery, Nanjing Drum Tower Hospital, The Affiliated Hospital of Nanjing University Medical School, Nanjing, China

**Keywords:** dilated cardiomyopathy, end-stage heart failure, biventricular assist device, volume management, case report

## Abstract

Approximately one-third of patients with end-stage heart failure (ESHF) experience biventricular failure. Left ventricular assist devices (LVADs) are employed as a salvage therapy for individuals with advanced heart failure. The onset of right ventricular failure complicates the postoperative management of patients receiving LVAD support. Currently, no right ventricular assist device is specifically designed for isolated right heart failure support, necessitating cardiac surgeons worldwide to adopt various improvised methods using LVADs for right ventricular assistance. This report details the first case at Nanjing Drum Tower Hospital in which a Corheart 6 magnetically levitated LVAD was successfully employed for biventricular assistance in a patient with ESHF secondary to dilated cardiomyopathy.

## Introduction

Patients with end-stage heart failure (ESHF) frequently present with severe conditions that significantly restrict daily activities, diminish quality of life, and increase mortality risk; however, conventional pharmacological treatments often have limited efficacy (1). Heart transplantation remains the gold standard for treatment but is hampered by a critical shortage of donor hearts. Consequently, ventricular assist devices (VADs) have emerged as essential tools for mechanical circulatory support (MCS) in the management of ESHF (2).

Right ventricular failure (RVF) commonly occurs as a secondary complication of prolonged left ventricular failure (LVF), although it may also arise from intrinsic pathology of the right ventricle itself (3). Literature suggests that 10%–30% of patients with ESHF experience biventricular failure (4). Data from the European Registry for Patients with Mechanical Circulatory Support (EUROMACS) indicated that among 3,282 patients, 413 (12.5%) required biventricular assist device (BiVAD) therapy (5). For patients who have contraindications to heart transplantation, BiVAD offers a viable long-term treatment option. Chronic LVF and systemic congestion may obscure the symptoms of RVF, making it challenging to identify RVF prior to the initiation of MCS. Following LVAD implantation, the normalization of left ventricular output increases venous return, thereby promptly revealing pre-existing RVF. If RVF persists despite volume adjustment and positive inotropic support, the implantation of a temporary or permanent RVAD becomes necessary. For ESHF patients with confirmed preoperative RVF and no contraindications, concurrent BiVAD implantation is recommended; similarly, if post-LVAD RVF occurs, timely RVAD implantation is also crucial.

Currently, no mechanical assist device fully conforms to the anatomical and physiological characteristics of the right ventricle. The Corheart 6 LVAD (Shenzhen Core Medical Technology Co., Ltd.) presents an option for off-label use as an RVAD. As the world's smallest and lightest LVAD, the Corheart 6 has demonstrated favourable blood compatibility and has been extensively implanted in children with ESHF in China (6). However, there are relatively few case reports regarding the use of the Corheart 6 for BiVAD support in China (7).

Since 2022, our center has successfully treated 20 patients with ESHF using the domestically manufactured third-generation magnetically levitated Corheart LVAD, achieving satisfactory clinical outcomes. One patient, who suffered from severe dilated cardiomyopathy resulting in biventricular failure, underwent concurrent LVAD and RVAD implantation, leading to hemodynamic optimization. This report retrospectively analyses and summarizes the assessment techniques, ideal BiVAD parameter settings, and complication management strategies employed in this case.

## Case presentation

A 41-year-old male of Han ethnicity, with a family history of dilated cardiomyopathy, presented with a height of 173 cm, weight of 60 kg, and body surface area of 1.7 m^2^. He had no comorbidities, such as hypertension or diabetes, and reported no history of psychiatric disorders or substance abuse; his psychosocial status was assessed as healthy. The patient experienced exertional dyspnea and chest tightness for one year, with worsening symptoms over the past fortnight. He exhibited intolerance to anti-heart failure medications, presenting with refractory hypotension during treatment in the cardiology department. Physical examination revealed marked cardiac enlargement, and a holosystolic murmur (Grade II) was auscultated at the apical region and the lower left sternal border. Consequently, he was referred to the cardiac surgery department on 24th February.

An electrocardiogram indicated sinus rhythm with T-wave changes in leads V5 and V6. Coronary angiography of all major epicardial vessels (left anterior descending, left circumflex, and right coronary arteries) was normal, thereby decisively excluding ischaemic cardiomyopathy. Echocardiography demonstrated significant global cardiac enlargement, with a left ventricular end-diastolic diameter (LVDd) of 70.8 mm and a severely reduced left ventricular ejection fraction (LVEF) of 17%, indicating severe left ventricular systolic dysfunction. Impairment of right ventricular systolic function was also observed, with a tricuspid annular plane systolic excursion (TAPSE) of 1.2 cm, a systolic velocity of the tricuspid annulus (*S*′) of 6 cm/s, and a fractional area change (FAC) of 19.8%. Moderate mitral and tricuspid regurgitation were noted. Chest CT scans revealed bilateral lung infiltrates and inflammatory changes. Cardiac MRI confirmed the presence of left ventricular fibrosis, with an LVEF of 14%. Right heart catheterisation demonstrated a cardiac index (CI) of 1.3 L/min/m^2^, a central venous pressure (CVP) of 10 mmHg, a pulmonary vascular resistance (PVR) of 2.73 Wood units, and a pulmonary artery pulsatility index (PAPi) of 1.4. The patient was diagnosed with biventricular dilated cardiomyopathy, ESHF, and acute exacerbation of chronic heart failure, with cardiac function classified as New York Heart Association (NYHA) Class IV and Interagency Registry for Mechanically Assisted Circulatory Support (INTERMACS) Class 4.

Preoperative optimisation involved positive inotropic support with dobutamine and milrinone, alongside diuresis using nesiritide and furosemide. One week later, a repeat echocardiogram indicated persistent global cardiac enlargement (LVDd 70.2 mm, LVEF 19%) and improved right ventricular systolic function (TAPSE 1.3 cm, *S*′ 7.6 cm/s, FAC 24%), although moderate mitral and tricuspid regurgitation persisted. Right heart catheterisation results indicated a CI of 0.9 L/min/m^2^, a CVP of 10 mmHg, and a PVR of 1.3 Wood units, with a PAPi of 1.6. The results of the preoperative examinations are summarised in Table 1.

**Table 1:** Preoperative echocardiographic and right heart catheterization examination results of the patient.

Echocardiographic examination	Hospital day 2	Hospital day 8
LAD (cm)	4.27	4.2
LVDd (cm)	7.08	7.02
LVDs (cm)	6.55	6.39
EF (%)	17%	19%
TAPSE (cm)	1.2	1.3
*S*′(cm/s)	6.9	7.6
FAC (%)	19.80%	24%
IVC (cm)	1.85	1.65
RA (cm)	4.65 × 5.65	4.5 × 5.55
Right heart catheterization examination		
CVP (mmHg)	10	10
RAP (mmHg)	15/7 (10)	13/7 (9)
RVP (mmHg)	41/5 (17)	45/9 (21)
PAP (mmHg)	47/33 (38)	41/25 (30)
PAWP (mmHg)	32	28
CO (L/min)	2.2	1.5
CI (L/min/m2)	1.3	0.9
PVR (wood)	2.73	1.3
PAPi	1.4	1.6
CVP/PAWP	0.31	0.32

CI, cardiac index; CO, cardiac output; CVP, central venous pressure; EF, ejection fraction; FAC, fractional area change; IVC, inferior vena cava; LAD, left atrial dimension; LVDd, left ventricular diastolic diameter; LVDs, left ventricular systolic diameter; PAP, pulmonary artery pressure; PAPi, pulmonary artery pulsatility index; PAWP, pulmonary artery wedge pressure; PVR, pulmonary vascular resistance; RA, right atrium; RAP, right atrial pressure; RVP, right ventricular pressure; *S*′, systolic velocity of tricuspid annulus; TAPSE, tricuspid annular plane systolic excursion.

**Figure 1:**
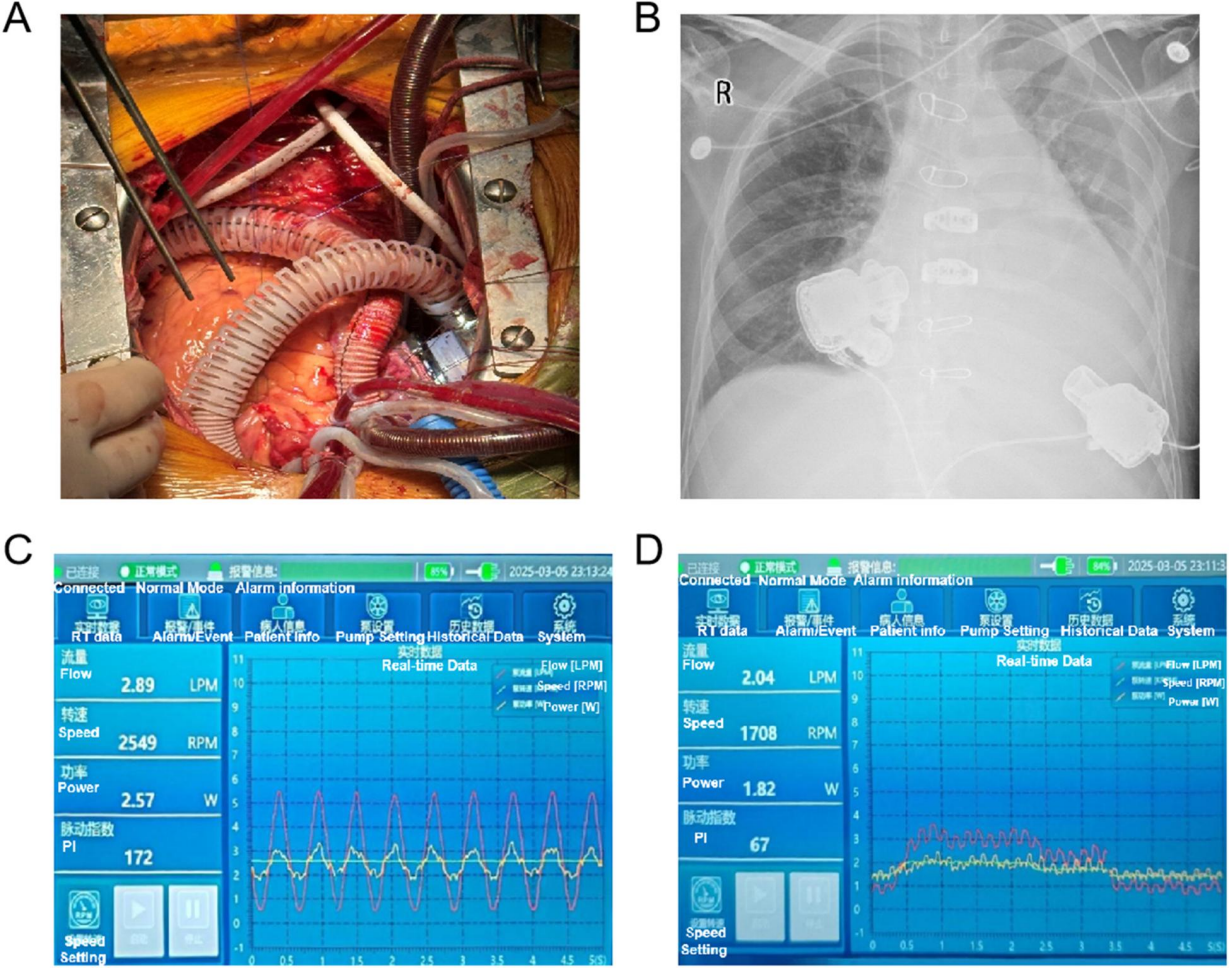
Intraoperative BiVAD implantation **(A)** and chest x-ray **(B)** in postoperative day 0 of the patient. **(C)** LVAD and **(D)** RVAD console interface displaying real-time hemodynamic parameters: flow rate, rotor speed, power, and pulsatility index (PI).

Following a multidisciplinary discussion involving cardiac surgery, anaesthesiology, cardiopulmonary bypass, cardiac surgery intensive care unit (CICU), and echocardiography teams, it was determined that the severe RVF in this patient may not be reversible with LVAD support (8). Consequently, the decision was made to proceed with BiVAD implantation on 5 March 2025, under general anaesthesia and cardiopulmonary bypass. Both the left and right ventricular assist devices employed the Corheart 6 implantable LVAD system, with the RVAD positioned in the right atrium (Figure 1). The operation was conducted with cardiopulmonary bypass (CPB) maintained for a duration of 220 min, during which satisfactory hemodynamic control was achieved. Intraoperative blood loss amounted to 2,000 ml, necessitating a transfusion of 1,700 ml of blood. Following the initiation of dual-pump operation, echocardiographic monitoring facilitated a gradual reduction of CPB flow, the synchronisation of LVAD flow increase, and a smooth transition from a “triple-heart” (left ventricle, cardiopulmonary bypass, and LVAD) to a “dual-heart” (LVAD and left ventricle) circulation state (Supplementary Figures 1A–D). The final settings were LVAD at 2,550 rpm with a flow rate of 4.5 L/min and RVAD at 1,700 rpm with a flow rate of 2.3 L/min.

Postoperatively, the patient was transferred to the CICU, with the LVAD at 2,549 rpm (2.89 L/min) and the RVAD at 1,708 rpm (2.04 L/min) (Figures 1C,D), maintaining mean arterial pressures (MAPs) around 75 mmHg. Comprehensive management included mechanical ventilation, optimization of cardiac preload and afterload, administration of positive inotropic drugs, dynamic adjustment of VAD parameters, support for organ function, infection control, anticoagulation, and maintenance of internal environment stability. During the patient's treatment in the CICU, daily bedside transthoracic echocardiography was performed by a same CICU physician, focusing on the assessment of the left and right hearts’ balance, aortic and pulmonary valve opening status, valvular regurgitation severity, as well as inferior vena cava diameter and its respiratory variability. Early postoperative hemodynamic indicators, VAD parameter settings, fluid balance, and laboratory results are detailed in Supplementary Table 1. The patient was successfully weaned off the ventilator and extubated on the first postoperative day after 16.5 h of mechanical ventilation. Sequential high-flow oxygen therapy (30% oxygen concentration, 45 L/min flow rate) was initiated, gradually tapering to 2 L/min oxygen by postoperative day 4, with a respiratory rate of 16–25 breaths/min and peripheral oxygen saturation of 98%–100%. Anticoagulation commenced on postoperative day 1 with unfractionated heparin, targeting an activated partial thromboplastin time (APTT) of 40–60 s, and was adjusted with warfarin to achieve an international normalized ratio (INR) of 2.5–3. By day 3, the INR reached the target value, and heparin was discontinued following continuous administration for 24 h.

**Figure 2:**
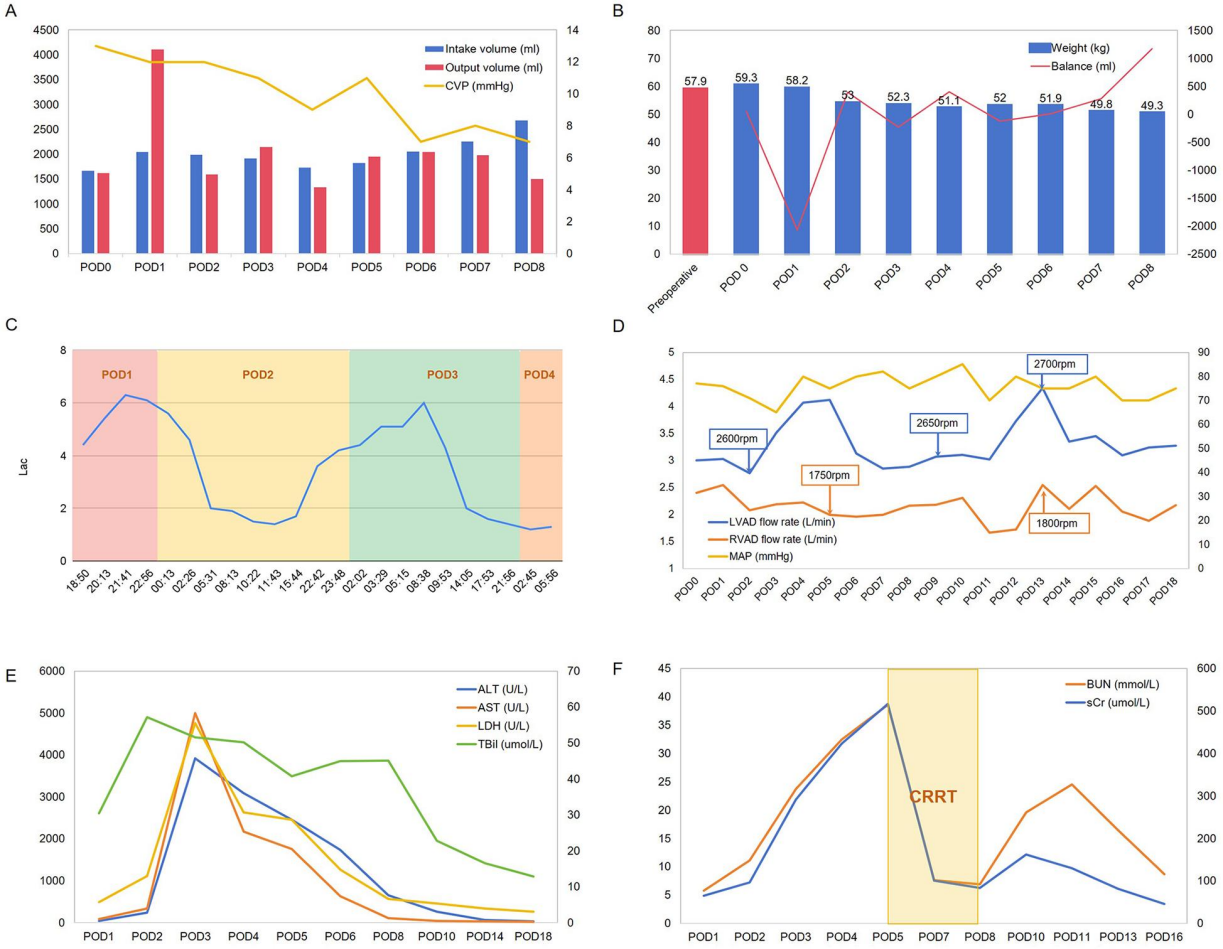
Hemodynamic parameters **(A)**, weight and fluid balance status **(B)**, Lac levels **(C)**, ventricular assist device settings **(D)**, and laboratory test results **(E,F)** of the patient during postoperative days (POD).

The patient's preoperative weight was 57.9 kg, while their postoperative weight upon returning to the CICU was 59.3 kg following surgery under CPB, with a CVP was 13 mmHg (Figures 2A,B). On postoperative day 1, transthoracic echocardiography revealed balanced left and right ventricular sizes, indicated by a left ventricle to left atrium (LV/LA) ratio of 1.63 and a left atrium to right atrium (LA/RA) ratio of 1.19. The aortic valve exhibited a 1:1 opening pattern, with an inferior vena cava diameter of 20 mm and a diameter variability of 19%. (Supplementary Figures 1E–H). We regulated the patient's fluid intake to approximately 2,000 ml. However, the patient experienced a significant increase in urine output on postoperative day 1, along with insensible fluid loss, resulting in an approximate weight loss of 5 kg within 24 h (Figure 2B).

On postoperative day 2, the patient's hepatic enzyme, creatinine, and blood urea nitrogen (BUN) levels increased significantly, accompanied by a decrease in MAP and elevated lactate (Lac) levels (Figures 2C,E,F). Given the potential for hypovolaemia to compromise tissue perfusion, we aimed to regulate net fluid output from postoperative days 2–5, striving to maintain a balance between fluid intake and output. Although the patient's liver enzymes showed a downward trend, BUN and serum creatinine levels continued to increase. Consequently, on postoperative day 5, we initiated continuous renal replacement therapy to facilitate toxin removal (Figure 2F), along with hepatoprotective, choleretic, and nephroprotective medications. By postoperative day 8, the patient's urine output had improved, allowing for discontinuation of the haemofiltration treatment. As the patient continued to recover, we increased fluid intake allowances and enhanced nutritional support, leading to a gradual return to preoperative weight, accompanied by declining levels of hepatic enzymes, bilirubin, and BUN. During this period, we also attempted to adjust the rotational speed of the BiVADs in response to reduced blood flow (Figure 2D).

Early rehabilitation commenced on postoperative day 1, involving bed limb exercises and ankle pump movements. By day 5, the patient progressed to sitting in a wheelchair and assisted ambulation at the bedside, ultimately achieving independent walking of approximately 70 m with a walker by day 16. On day 19, the patient was transferred to the general ward. Echocardiography performed on postoperative day 13 demonstrated reduced cardiac chamber sizes compared to the preoperative examination, with balanced left and right heart sizes, properly functioning aortic and pulmonary valves exhibiting a 1:1 opening pattern (with mild regurgitation), and significantly diminished mitral and tricuspid regurgitation (also mild) (Supplementary Figures 1I–L).

**Figure 3:**
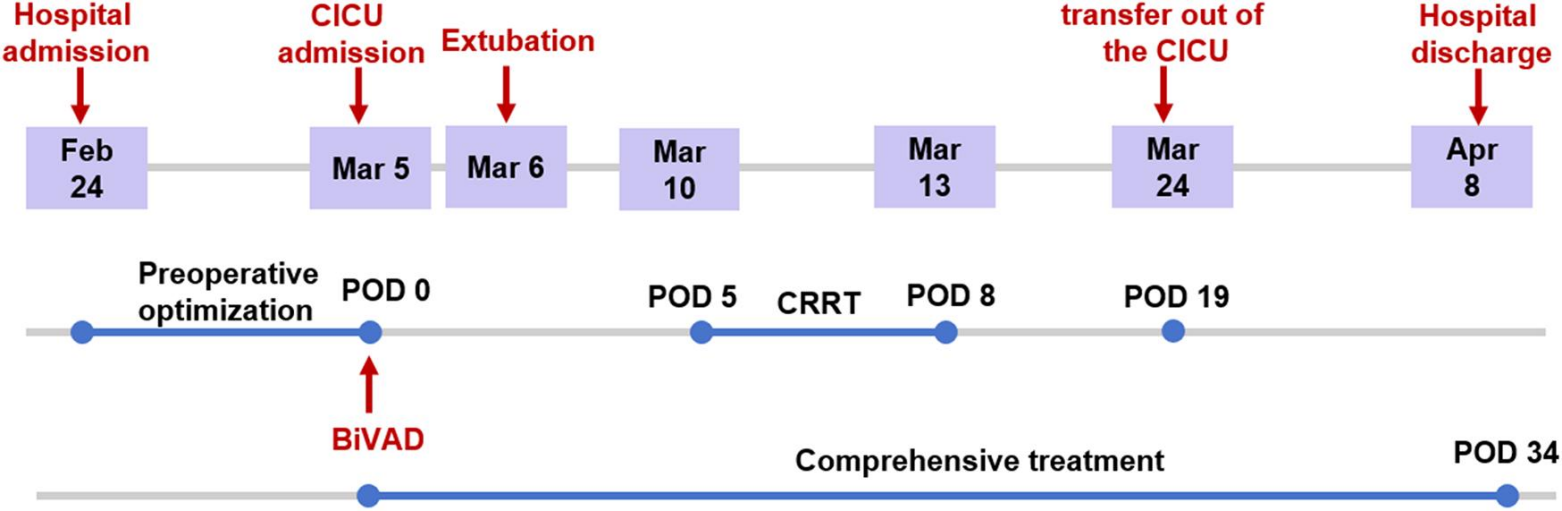
Timeline of treatment of the patient.

The patient was discharged on postoperative day 34. Figure 3 illustrates the timeline of the patient's treatment. After discharge from the hospital, he continues regular administration of warfarin and aspirin for thromboprophylaxis, along with ongoing anti-heart failure medications including diuretics, digoxin, metoprolol, Entresto (sacubitril/valsartan), vericiguat, and empagliflozin.

During a two-month follow-up, the patient exhibited stable haemodynamic, preserved organ function, and no thrombotic or bleeding complications. The patient expressed high satisfaction with the treatment process, noting substantial relief from symptoms such as chest tightness and dyspnoea, as well as significant improvements in quality of life, including restored mobility and daily functioning, and emotional relief following the successful implantation of the BiVAD.

## Discussion

Biventricular failure is a common complication in patients with ESHF, occurring in approximately 30% of cases. The rapid advancement of ventricular mechanical assist technology over the past two decades has established left heart assist implantation as a highly effective alternative therapy for patients with ESHF. In individuals receiving LVADs, RVF significantly complicates postoperative recovery, necessitating temporary or permanent RVAD support in approximately 10% of recipients (9–11). LVADs alleviate the workload of the left ventricle, thereby reducing left atrial pressure and enhancing pulmonary artery compliance, which in turn alleviates right ventricular afterload. However, in patients with independent RVF, LVAD implantation can disrupt the equilibrium between left and right ventricular outputs, consequently exposing and exacerbating RVF. Currently, no dedicated persistent RVAD exist, leading to the off-label use of small continuous-flow LVADs as RVADs, a strategy gaining international recognition (12).

Left ventricular assist devices are classified into three generations based on their operational principles: the first generation with pulsatile blood flow, the second generation with continuous blood flow and mechanical bearings, and the third generation with blood pumps featuring magnetic levitation and contactless bearings. The current global standard for left heart failure treatment is provided by centrifugal pumps (e.g., HeartMate III), although axial flow pumps like the HeartMate II also represent significant earlier technology (13, 14).

INTERMACS data covering the period from 2006 to 2016 revealed 349 pulsatile BiVADs (1.5%) and 616 continuous-flow BiVADs (2.7%) among 22,866 implanted devices (15). By 2019, continuous-flow BiVADs had largely supplanted pulsatile devices, accounting for 3.9% of long-term MCS implants (16). An early case report in 2004 by Radovancevic et al. documented the first successful biventricular support using dual Jarvik 2,000 devices (17). Shortly thereafter, German research teams reported achieving similar biventricular assistance through the application of HeartWare Ventricular Assist Device systems (18, 19). The Corheart 6 LVAD (Shenzhen Core Medical Technology Co., Ltd.), a third-generation implantable left heart support device featuring an axial magnetic levitation design, provides continuous-flow mode, offers the advantages of improved myocardial function and adjustable blood flow, thus presenting a viable option for off-label use as a RVAD. As the world's smallest and lightest LVAD, the Corheart 6, which boasts favourable blood compatibility, has been widely implanted in children with ESHF in China (6). However, the characteristics of the continuous-flow mode also change the original pathophysiological mechanisms of the heart, posing several challenges to postoperative management, including but not limited to difficulties in accurately assessing fluid management (due to the altered hemodynamics), potential risks of thrombus formation associated with the non-pulsatile flow, challenges in maintaining appropriate anticoagulation levels, and the need for specialized monitoring and adjustment strategies to ensure optimal organ perfusion and prevent complications related to the unique flow dynamics of continuous-flow devices.

In this case report, we present the first successful instance of BiVAD treatment utilising the Corheart 6 system at our centre, which not only validates the feasibility of its clinical application in managing adult ESHF in China but also provides invaluable insights for future research.

INTERMACS data reveal a concerning one-year survival rate of 56% for BiVAD recipients (20). The primary causes of mortality included multisystem organ failure (43%) and sepsis (13%), followed by stroke and bleeding events (21). A multicentre study involving 14 patients supported by the fully magnetically levitated centrifugal pump HeartMate III as BiVADs reported five fatalities occurring on days 10, 60, 83, 99, and 155. These deaths were attributed to sepsis (three cases), haemorrhagic stroke (one case), and RVAD thrombosis (one case). Additional complications included one case of pump thrombosis requiring replacement, two cases of sepsis, and one case each of renal failure, gastrointestinal bleeding, and epistaxis (22). Several factors may contribute to the poor prognosis of patients receiving BiVADs (20, 22): (1) these patients are typically critically ill, predominantly classified as INTERMACS level 1 or 2; (2) preoperative reliance on temporary rotational pumps or extracorporeal membrane oxygenation (ECMO) for right ventricular support; (3) the absence of dedicated RVADs; (4) insufficient expertise in RVAD implantation and management; and (5) for some patients with low cardiac output and a small thoracic cavity, the unavailability of appropriately sized VADs presents a significant challenge. Consequently, minimising and promptly addressing complications are primary objectives in postoperative management. The patients in this study exhibited typical characteristics of biventricular failure and demonstrated a poor response to conventional pharmacological treatments, thereby meeting the criteria for BiVAD implantation. This patient's preoperative NYHA classification was Grade IV and INTERMACS Class 4, and there was no evidence of irreversible multiple organ failure, which created favourable conditions for the success of the procedure.

Several indices, primarily derived from right heart catheterisation and echocardiographic assessments, have been employed to predict the occurrence of RV failure following LVAD implantation (23). These indices include elevated right atrial pressure (RAP > 15 mmHg) as a marker of increased RV preload (24, 25), low mean pulmonary artery pressure (mPAP) combined with impaired RV systolic function, and increased PVR {[mean PAP—mean pulmonary capillary wedge pressure (PCWP)]/cardiac output} (26, 27). Additionally, an RAP/PCWP ratio > 0.63 (28, 29) and a low pulmonary artery pulsatility index (PAPi) [(pulmonary systolic artery pressure—pulmonary diastolic artery pressure)/RAP] < 1.85 (30) have been identified as risk factors for right heart failure. Echocardiographic parameters such as TAPSE (which lacks consistent prognostic value) (31), the peak systolic velocity of the tricuspid annulus (ranging from 8.0 to 8.8 cm/s, although data are conflicting) (25, 32), and impaired RV FAC have shown predictive potential (31). In this case, the patient exhibited multiple impaired markers of RV dysfunction preoperatively, with no signs of RV function improvement despite all attempts at RV optimisation. Given the patient's increased risk for developing RV failure post-LVAD implantation, we planned for biventricular circulatory support from the outset. In this case, the implantation site for the RVAD was selected to be the right atrium. This strategy has been demonstrated to be feasible in certain international studies and may reduce surgical complexity while enhancing safety (7, 33).

The appropriate matching of LVAD and RVAD parameters is crucial post-BiVAD implantation. With the RVAD positioned in the right atrium, excessive flow can completely decompress the right ventricle, leading to ventricular stasis and an increased risk of thrombosis. Research indicates that non-pulsatile RVAD flows exceeding 4 L/min are associated with pulmonary haemorrhage (34). Consequently, adjustments to RVAD speed and flow should ensure partial filling of the right ventricle, supported by positive inotropic agents, to maintain right ventricular contractility. This approach facilitates a dual blood flow pattern into the pulmonary artery from both the RVAD and right ventricle, augmenting pulmonary circulation and left ventricular preload. If the LVAD speed is not increased concurrently, elevated pulmonary venous pressure may lead to pulmonary oedema. In accordance with established guidelines, our centre sets BiVAD speeds to achieve a left ventricular assist pump CI of at least 2.2 L/min/m^2^ and modulates RVAD speed based on CVP, ensuring that LVAD flow exceeds RVAD flow by 5%–10% (35). Simultaneously, excessive LVAD speed and flow are avoided to prevent elevated aortic pressures, reduced aortic valve opening, and subsequent aortic insufficiency due to valve fusion, which can render LVAD support ineffective. Our centre's experience suggests that intraoperative pump speed adjustments, guided by echocardiography, should aim for effective decompression of both the left and right ventricles, maintaining CVP < 14 mmHg and pulmonary artery wedge pressure between 8 and 13 mmHg, with a centrally positioned interventricular septum and adequate tissue perfusion. A low-speed, low-flow strategy is preferred to preserve partial right ventricular function and aortic valve opening, ensuring that LVAD flow consistently exceeds RVAD flow by 5%–10%.

Post-BiVAD implantation, patients’ volume status significantly influences the balance of the left and right heart and overall tissue perfusion. Volume overload can elevate venous pressure, hinder venous return, and lead to tissue oedema and organ congestion, while volume depletion diminishes cardiac output and tissue perfusion. Achieving an optimal volume balance is pivotal for maintaining circulatory stability, adequate tissue perfusion, and normal organ function. Recipients of BiVADs frequently exhibit considerable volume overload and tissue oedema postoperatively, complicating accurate volume assessment due to the absence of a single, definitive clinical measurement. A comprehensive evaluation incorporating CVP, PCWP, chest radiography, echocardiography, daily weight, and tissue oedema is essential. Despite the initial weight gain upon admission to the CICU following CPB, the patient subsequently developed signs of circulatory instability on postoperative days 2–3, including hypotension, decreased CVP, and elevated lactate levels, accompanied by acute hepatic and renal dysfunction. Renal dysfunction is also common after LVAD surgery and is associated with increased postoperative mortality. It is generally believed that the main causes include alterations in renal perfusion, systemic activation of oxygenation, and the inflammatory cascade response (36). Additionally, the use of nephrotoxic drugs for infection prevention can adversely affect renal function. In our study, although the impact of surgery on CPB regarding hepatic and renal function requires consideration (37), the other manifestations in this patient necessitate attention to inadequate tissue perfusion resulting from hypovolemia (38, 39). This underscores the critical importance of volume management in the postoperative recovery of patients undergoing BiVAD implantation. The CorHeart 6 system's continuous-flow design alters cardiac hemodynamic patterns, theoretically compromising the reliability of dynamic fluid responsiveness indices that depend on normal cardiac pulsatile flow, such as pulse pressure variation (PPV) and stroke volume variation (SVV). These indices still have clinical reference value when the aortic valve opens in a 1:1 synchrony pattern, but their utility also limited by both peripheral vascular reactivity and under mechanical ventilation. Therefore, in clinical practice, a comprehensive assessment should integrate pump parameters (such as rotational speed and power), echocardiography, the passive leg-raising test, blood lactate levels, CVP, body weight, and other factors. Considering the early stage of biventricular assist device implementation in China, its unique hemodynamic profile demands tailored fluid management strategies. Future research should focus on developing device-specific evaluation systems for precise fluid therapy, such as algorithm models based on pump parameters.

An individualized anticoagulation strategy is critical for ensuring a patient's postoperative recovery. The low-pressure, low-resistance pulmonary circulation, combined with relatively lower speeds of RVAD, increases the risk of pump thrombosis, with reported rates as high as 37% following BiVAD implantation (21, 40). In accordance with established guidelines, our protocol includes early heparin bridging to warfarin therapy, targeting an INR of 2.5–3, and initiating oral aspirin (100 mg/day) once platelet counts exceed 100 × 10^9^/L (35). The patient achieved the target INR level by postoperative day 3. Some studies suggest that the INR should be maintained between 2.0 and 2.5 (41). This regimen is convenient and manageable, providing a superior anticoagulation advantage for the Corheart 6 LVADs. Furthermore, as an implantable mechanical assist device, postoperative bleeding remains a significant concern. The dual-pump, high-flow configuration, combined with multiple wound sites and increased shear forces, elevates the risk of postoperative bleeding (21). In this study, the patient did not experience pump thrombosis, major bleeding, or hemolysis. This can be attributed to the internal fluid path design of the blood pump, which reduces the surface area of blood contact, shear stress within the pump, and blood residence time, thereby minimising blood “damage” and enhancing blood compatibility.

This study presents a successful case of BiVAD therapy employing the Corheart 6 magnetically levitated LVAD for the treatment of ESHF secondary to dilated cardiomyopathy, achieving satisfactory short-term clinical outcomes. However, this study had some limitations. First, this report is based on a single-patient experience, which inherently introduces selection bias and limits the generalisability of our findings. Our centre is currently enrolling additional patients undergoing BiVAD therapy to validate its efficacy and safety. Second, while this case report offers detailed documentation of short-term postoperative recovery, it lacks longitudinal data on long-term survival and quality of life outcomes. Further follow-up research is necessary to evaluate long-term survival rates and quality of life for these patients. Third, as there is currently no dedicated persistent RVAD, the RVAD in this study was an off-label use of small continuous-flow LVADs, which may introduce suboptimal haemodynamic matching and potential durability concerns. Future studies should explore innovative directions in RVAD technology, including the development of more compact and intelligent device designs, as well as new technologies, such as wireless power transmission.

In conclusion, while BiVAD implantation is associated with high complication and mortality rates; appropriate LVAD and RVAD speed and flow settings, coupled with meticulous volume status management, optimization of cardiac preload and afterload, individualized anticoagulation strategies, and vigilant organ function monitoring can successfully facilitate perioperative recovery. Our case demonstrates that biventricular assist implantation remains a highly effective alternative to heart transplantation for treating end-stage global heart failure.

## Data Availability

The original contributions presented in the study are included in the article/Supplementary Material, further inquiries can be directed to the corresponding authors.
