# Supplementary material for: A case report: corheart 6 biventricular assist device therapy for end-stage heart failure in dilated cardiomyopathy
Source: Front Cardiovasc Med. 2025 Oct 28;12:1677940. doi: 10.3389/fcvm.2025.1677940 (PMC12602535; doi:10.3389/fcvm.2025.1677940)
Supplement: Supplementary file 1 [file Datasheet1.pdf]

A Case Report: Corheart 6 Biventricular Assist Device Therapy for End-Stage Heart Failure in Dilated Cardiomyopathy

Qiuju Ding[#1], Cheng Chen[#1], Zhenjun Xu, Ning Zhang, Jun Pan[*1], Min Ge[*1]

[1]Department of Cardio-thoracic Surgery, Nanjing Drum Tower Hospital, The Affiliated Hospital of Nanjing University Medical School, Nanjing, China.

[#]These authors contributed equally.

***Corresponding author:**

Jun Pan, Ph.D., M.D.

Department of Cardio-thoracic Surgery, Nanjing Drum Tower Hospital, The Affiliated Hospital of Nanjing University Medical School, Zhongshan Road 321, Nanjing 210008, China.

E-mail: pj791028@163.com

Min Ge, Ph.D., M.D.

Department of Cardio-thoracic Surgery, Nanjing Drum Tower Hospital, The Affiliated Hospital of Nanjing University Medical School, Zhongshan Road 321, Nanjing 210008, China.

Email: gemin2000@outlook.com

Supplementary Figure 1. Animated illustrations of echocardiographic examinations during surgery (A-D), on postoperative day 1 (E-H), and on postoperative day 13 (I-L).

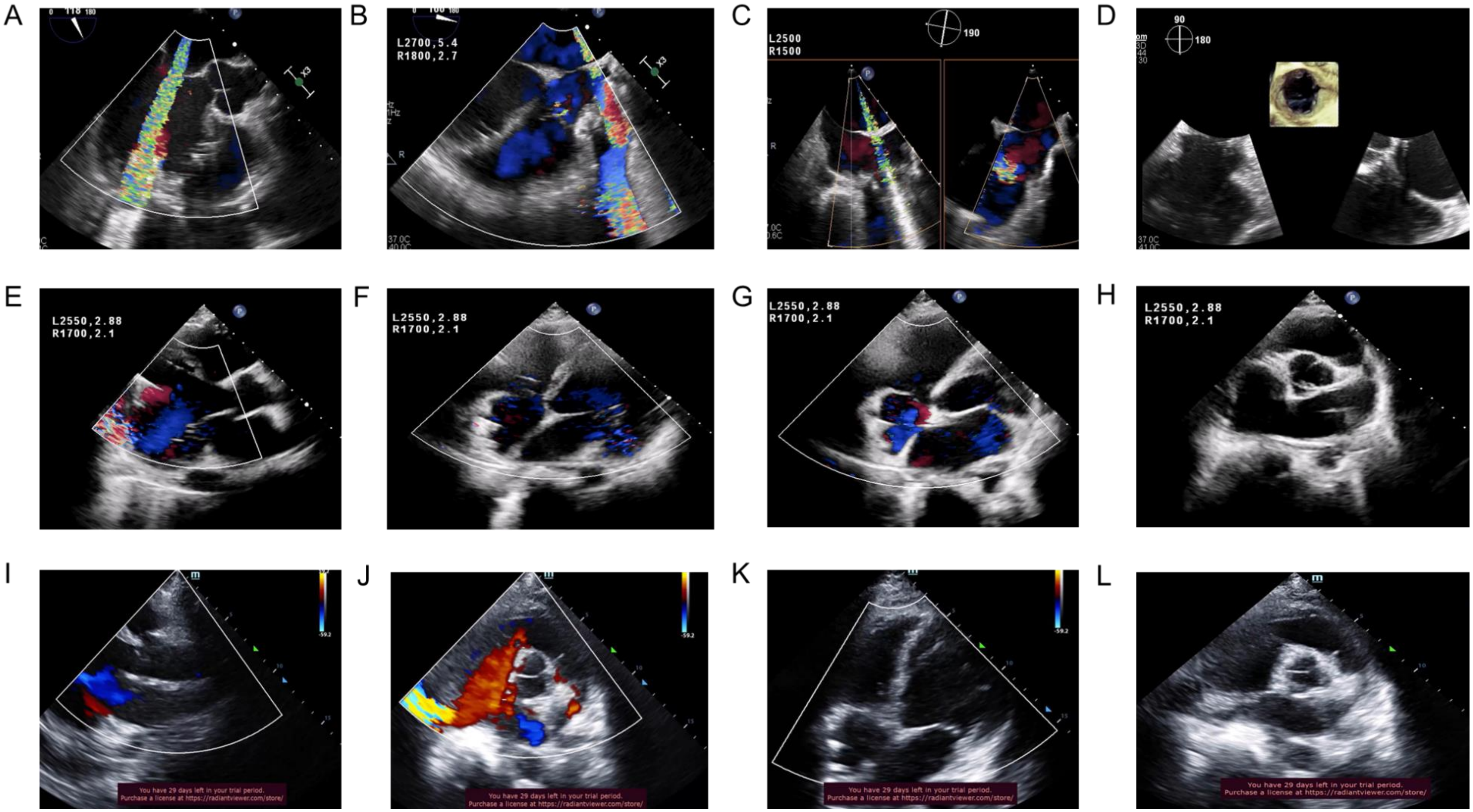

Supplementary Table 1. Hemodynamic parameters, ventricular assist device settings, fluid balance, and laboratory test results of the patient from postoperative day 1 to day 18.

Days	Intake (ml)	Output (ml)	Balance (ml)	Weight (kg)	LVAD speed (rpm)	LVAD flow rate (L/min)	RVAD speed (rpm)	RVAD flow rate (L/min)	MAP (mmHg)	CVP (mmHg)	Lac (mmol/L)	ALT (U/L)	AST (U/L)	sCr (umol/L)	BUN (umol/L)
**POD 0**	1671	1625	46	59.3	2549	3	1665	2.4	77	13	1.8	/	/	/	/
**POD 1**	2044	4111	-2067	58.2	2549	3.03	1737	2.54	76	12	6.3	41.9	86.6	65	5.8
**POD 2**	1987	1594	393	53	2549	2.76	1696	2.08	71	12	5.1	239	336	96	11.1
**POD 3**	1914	2144	-230	52.3	2598	3.51	1726	2.19	65	11	6	3920	4998	291	23.7
**POD 4**	1732	1331	401	51.1	2598	4.07	1706	2.22	80	9	1.4	3085	2170	423	32.4
**POD 5**	1827	1953	-126	52	2598	4.12	1750	1.99	75	11	1.8	2455	1760	516	38.6
**POD 6**	2057	2047	10	51.9	2598	3.13	1816	1.96	80	7	1.5	1734	634	/	/
**POD 7**	2254	1976	278	49.8	2595	2.85	1763	1.99	82	8	1.9	/	/	101	7.6
**POD 8**	2677	1505	1172	49.3	2598	2.88	1723	2.16	75	7	2.1	656	114	83	6.9
**POD 9**	3657	3200	457	52.3	2648	3.07	1701	2.18	80	8	1.2	/	/	/	/
**POD 10**	2611	1930	681	52.7	2648	3.10	1802	2.31	85	10	1	262	42.1	162	19.6
**POD 11**	2545	1960	585	52.7	2648	3.02	1801	1.66	70	8	1.5	/	/	130	24.5

**POD 12**	2646	1730	916	53.4	2648	3.73	1799	1.72	80	9	1.8	/	/	/	/
**POD 13**	2572	2630	-58	54	2698	4.34	1831	2.54	75	7	1.6	/	/	81	16.4
**POD 14**	2494	2450	44	55.5	2698	3.35	1866	2.1	75	10	1.3	68.4	30.4	/	/
**POD 15**	2431	2990	-559	56.9	2698	3.45	1753	2.53	80	9	1.6	/	/	/	/
**POD 16**	2672	2890	-218	58.2	2698	3.09	1820	2.05	70	7	1.6	/	/	46	8.7
**POD 17**	2815	3430	-615	58.8	2698	3.24	1808	1.88	70	9	1.8	/	/	/	/
**POD 18**	2622	2900	-278	59.4	2698	3.27	1795	2.17	75	8	1.3	37	24.1	/	/

Abbreviations: ALT: alanine aminotransferase; AST: aspartate aminotransferase; BUN: blood urea nitrogen; CVP: central venous pressure; Lac: lactate; LVAD: left ventricular assist device; MAP: mean arterial pressure; POD: postoperative day; RVAD: right ventricular assist device; sCr: serum creatinine.
